# Seroprevalence and Risk Factors of* Toxoplasma gondii* Infection among Domestic Ruminants in East Hararghe Zone of Oromia Region, Ethiopia

**DOI:** 10.1155/2018/4263470

**Published:** 2018-05-20

**Authors:** Berhanu Tilahun, Yacob Hailu Tolossa, Getachew Tilahun, Hagos Ashenafi, Shihun Shimelis

**Affiliations:** ^1^Department of Parasitology, College of Veterinary Medicine, Haramaya University, P.O. Box 138 Dire Dawa, Ethiopia; ^2^Department of Pathology and Parasitology, College of Veterinary Medicine and Agriculture, Addis Ababa University, P.O. Box 34, Bishoftu, Ethiopia; ^3^Aklilu Lemma Institute of Pathobiology, Addis Ababa University, P.O. Box 1176, Addis Ababa, Ethiopia

## Abstract

A cross-sectional study was carried out from July 2011 to September 2013 to assess the seroprevalence and identify risk factors of* Toxoplasma gondii* infection in domestic ruminants of East Hararghe zone of Oromia region, Ethiopia. Sera of 1360 domestic ruminants were analyzed for the presence of anti-*T. gondii* IgG antibodies using the indirect enzyme-linked immunosorbent assay (iELISA). Additionally, the owners were also interviewed using a structured questionnaire to identify the potential risk factors of* T. gondii* infection. Overall, the prevalence of* T. gondii* infection in domestic ruminants was 22.2% (302/1360). The seroprevalence in sheep, goats, cattle, and camels was 33.7%, 27.6%, 10.7%, and 14.4%, respectively. District, species, sex, age, and water source were identified as risk factors for* T. gondii* infection. Increased seropositivity was observed in females (OR = 2.63) and also with the use of pond (OR = 4.25) and pipe (OR = 9.57) water sources in sheep; age >1 year old (OR = 3.45) and with drinking from pond (OR = 6.03) and pipe (OR = 11.61) water sources in goats; with the use of pond (OR = 5.60) and pipe (OR = 10.68) water sources in cattle; and in >4-year-old camels (OR = 2.49). In conclusion,* T. gondii* infection is common and widespread among the domestic ruminants of the study area, indicating the potential transmission to humans from these animals when they are used as a source of food. Hence, it is crucial to raise awareness of the people about* T. gondii* infection and conduct further study to explore the impact of the disease on food animal production.

## 1. Introduction

*Toxoplasma gondii* is an obligate intracellular protozoan parasite with worldwide distribution which can infect all warm blooded animals and man. Most animals and man serve as intermediate hosts, while domestic cats and wild felids are definitive hosts that have an important role in the transmission of* T. gondii* infection by shedding oocysts to the environment. Animals and humans can acquire* T. gondii* infection by ingesting feed and water contaminated with cats' faeces containing infective oocysts and animal tissues with viable cysts of* T. gondii* and congenitally [1].

Toxoplasmosis is an economically important disease of livestock, causing abortion, stillbirth, fetal malformation, preterm deliveries, and neonatal death predominantly in sheep and goats infected by* T. gondii*, subsequently creating a potential challenge to the small ruminant industry worldwide [2]. Food animals infected with* T. gondii* pose a risk for the public, since consumption of raw or undercooked meat from such animals can facilitate zoonotic transmission. Moreover,* T. gondii* can be congenitally transmitted to the foetus during pregnancy [1, 3, 4].

Several reports have indicated a great variation on the seroprevalence of* T. gondii* infection in domestic ruminants across the world and it ranges from 3% to 92% in sheep [3, 5], from 5% to 77% in goats [3, 6, 7], and from 0% to 99% in cattle [8]. In Africa, the reported seroprevalence in animals varied between 3.6% and 57.5% [9–13].

The estimated domestic ruminant population of Ethiopia in millions is sheep (27.35), goats (28.16), cattle (55.03), and camels (1.10) [14]. These large populations of domestic ruminants are mostly raised for milk, meat production, and breeding purpose. The seroprevalence of* T. gondii* infection in Ethiopia ranges from 22.9% to 54.7% in sheep and from 11.6% to 74.8% in goats [15–20]. Aside from that, the only report found on the seroprevalence* T. gondii* infection in cattle was that of 6.6% in Central Ethiopia [16]. Nonetheless, to date, there is no report from East Hararghe zone. Therefore, this study was carried out to bridge this information gap with the objectives of determining the seroprevalence and identifying the potential risk factors of* T. gondii* infection in domestic ruminants of East Hararghe zone.

## 2. Materials and Methods

### 2.1. Study Area

The study was conducted in three selected districts of East Hararghe zone of Oromia region, Ethiopia. The districts were found at an altitude ranging from 950 to 2950 meters above sea level (masl). Accordingly, Gursum district is located at 9°7′–32°14′N latitude and 42°17′–42°38′E longitude with an altitude range of 1200–2950 masl; Babille district is located at 8°9′–9°23′N latitude and 41°16′–41°46′E longitude with an altitude ranging from 950 to 2000 masl; and Haramaya district is located at 9°9′–9°32′N latitude and 41°50′–42°05′E longitude with an altitude ranging from 1600 to 2140 masl. The districts are inhabited mainly by the “Oromo” tribe. Crop production and livestock rearing are the main economic activities in the rural areas, while small-scale trade is practiced in urban areas. Sorghum, maize, groundnut,* “chat” (“khat”)*, and wheat are the main crops produced [21].

### 2.2. Study Animals and Design

The study animals comprised indigenous sheep, goats, cattle, and camels kept under extensive grazing system. Districts were selected purposively depending on their accessibility. Within the districts, 38* “kebeles”* (lowest administrative units) from both rural and urban areas were identified by simple random sampling from the lists obtained from the respective district administrations.

The sample size was determined using a method recommended by Thrusfield [22]. A total of 1360 animals (sheep = 332, goats = 410, cattle = 326, and camels = 292) were sampled based on expected prevalence of 57.4% for sheep and 26% for goats [18] and 50% for both cattle and camels at 5% absolute precision and 95% confidence level. The sample size was allocated proportionally to the selected study districts. Individual sample units were identified systematically at night resting places, grazing areas, and watering points. Only sheep, goats, and cattle above six months of age and camels above two years of age were included in the study.

### 2.3. Blood Samples Collection and Serology

A total of 1360 animal sera were collected and analyzed from July 2011 to September 2013. Approximately 5–10 ml of blood was drawn from the jugular vein using plain vacutainer tubes and kept overnight at room temperature to clot for serum separation. Aliquots of sera were obtained by centrifugation at 3000 revolutions per minute for 10 min and the sera were transported to the parasitology laboratory of College of Veterinary Medicine and Agriculture at Bishoftu (Debre Zeyit) in ice box and stored at −20°C until they were tested.

All sera were tested for anti-*T. gondii* IgG antibodies using indirect ELISA multispecies diagnostic kit (ID VET Innovative Diagnostic, ID Screen, Montpellier, France). On each working day, only the required amount of antigen and sera were thawed prior to serological testing. The preparation of the reagents and the iELISA test were performed based on the manufacturer's recommendation at the National Animal Health Diagnostic and Investigation Center (NAHDIC) Sebeta, Ethiopia. Positive and negative controls were included in each test and an animal was considered to be infected when the serum presented an OD% ≥ 50% with ELISA. Herds or flocks were considered as* T. gondii* seropositive, when at least one animal was tested positive.

### 2.4. Questionnaire Survey

100 verbally consented owners of domestic ruminants whose herds and flocks were involved in the survey were interviewed using a structured close-ended questionnaire. The gathered information included the demographic characteristics of the animals such as age, sex, breed, herd size, and physiological status of the animals and their husbandry including house types, feed storage facilities, grazing types, and water sources as well as cats holding and the presence of feral cats in the vicinity (variables obtained from literatures).

### 2.5. Statistical Analysis

The data were analyzed using STATA version 11.0 for MA Windows (Stata Corp., College Station, USA). The data were categorized to make the analysis easy. Accordingly, sheep and goats were classified in two groups as ≤1 year and >1 year old, cattle into three groups as ≤1 year, >1–5 years, and >5 years old, and camels into two groups, ≤4 years and >4 years old. The flock/herd size for sheep, goats, and cattle was considered as small (<10 animals) or large (≥10 animals), while for camels it was considered as small (≤34 animals) or large (>34 animals). Type of housing with total confinement is categorized as pen for sheep and goats and barn for cattle, while it is classed as fence for those with access to the outside. Water source was classified as mixed (river, stream, pond, and well), pond, and pipe water (except camels). Species seroprevalence and the association of risk factors as independent categorical variable with* T. gondii* seropositivity were analyzed by the chi-square test (*χ*^2^). Logistic regression analysis was performed to measure the strength of association between the potential risk factors and* T. gondii* seropositivity. Noncollinear variables with values of *P* < 0.20 in univariable analysis at 95% confidence level were entered into multivariable logistic regression model. The level of statistical significance was set as *P* < 0.05.

## 3. Results

### 3.1. Serological Findings

Out of 1360 sera of domestic ruminants tested, the presence of anti-*T. gondii* IgG antibodies was detected in 302 (22.2%, 95% CI: 20.0%–24.5%) of them that belonged to 173 (49.29%, 95% CI: 43.94%–54.65%) herds or flocks. The highest seroprevalence of infection was observed in sheep (33.7%) and the lowest was in cattle (10.7%). The distribution of* T. gondii* infection among domestic ruminants is presented in Table 1.* T. gondii* infection was detected in 32 out of the 38 (84.2%) farm areas/*“kebeles”* included in the study.

In a univariable analysis, goats had high risk of* T. gondii* infection (OR = 0.75, 95% CI: 0.55–1.02, *P* = 0.069) comparable with that of sheep, whereas cattle (OR = 0.24, 95% CI: 0.16–0.36, *P* < 0.001) and camel (OR = 0.33, 95% CI: 0.22–0.49, *P* < 0.001) were at low risk.

#### 3.1.1. Sheep

A multivariable logistic regression analysis showed that sex of the animal and water source were risk factors for increased seroprevalence. A higher risk of* T. gondii* infection occurred in females (OR = 2.63, 95% CI: 1.18–5.88, *P* = 0.019) than in males in those given pipe water (OR = 9.57, 95% CI: 5.00–18.33, *P* < 0.001) and pond water (OR = 4.25, 95% CI: 2.15–8.38, *P* < 0.001) compared with those that drank from a mixed water source (Table 2). But the variables district, age, and cats contact were insignificant (*P* > 0.05). Nonetheless, relatively higher seroprevalence was observed in sheep from Babille district (45.7%) than from Gursum (30.8%) and Haramaya (26.3%), in >1 year old (35.2%) than in ≤1 year old (23.1%), and in those having contact with cats (40.0%) than in those not in contact (30.0%).

#### 3.1.2. Goats

District, age, and water sources were identified as risk factors for* T. gondii* infection. An increased risk of infection occurred in goats >1 year old (OR = 3.45, 95% CI: 1.34–8.90, *P* = 0.010) compared to ≤1 year and in those given pipe (OR = 11.61, 95% CI: 4.35–30.95, *P* < 0.001) and pond water (OR = 6.03, 95% CI: 2.42–15.05, *P* < 0.001) than in those that drank from mixed water sources. In contrast, Babille district (OR = 0.15, 95% CI = 0.05–0.48, *P* < 0.001) was found to be a lower risk for goats (Table 3). Variables such as sex, breed, and flock size were not found to be significant (*P* > 0.05). However, raised seroprevalence was obtained in females (30.6%) compared to males (17.2%) and in small flock size (29.3%) compared to large (20.9%).

#### 3.1.3. Cattle

Multivariable logistic regression analysis indicated that district, herd size, and water source were risk factors for* T. gondii* infection. Using pond water (OR = 5.60, 95% CI: 2.12–14.78, *P* < 0.001) and pipe water sources (OR = 10.68, 95% CI: 2.23–51.22, *P* = 0.003) had significantly increased the risk of acquiring* T. gondii* infection compared to using mixed water sources, while living in Gursum district (OR = 0.19, 95% CI: 0.06–0.59, *P* = 0.004) and belonging to large herd size (OR = 0.35, 95% CI: 0.13–0.97, *P* = 0.044) were associated with lower risk (Table 4). Among the variables included in the statistical analysis, cats contact and house types were not significant (*P* > 0.05).

#### 3.1.4. Camels

The sole risk factor identified in camels is age. Camels in the age group of >4 years (OR = 2.49, 95% CI: 1.14–5.45, *P* = 0.022) showed an increased risk of* T. gondii* infection than those ≤ 4 years (Table 5). Although not statistically significant, difference seen (*P* > 0.05) in* T. gondii* infection in camels having contact with cats (21.7%) was higher than that seen in those not having contact (13.0%).

## 4. Discussion

The present study had given an insight on toxoplasmosis and revealed the widespread occurrence of* T. gondii* infection among the domestic ruminants raised in East Hararghe zone. The observed seroprevalence of* T. gondii* infection in sheep in this study is in agreement with those previously reported from Ethiopia, 31.59% from East and West Shewa Zones of Oromia Region [15] and 34% in Debre Birhan [17], and from elsewhere in the world, 29.41% and 32.9% from Brazil [5, 23] and 27.6% from Morocco [9]. But the current prevalence is slightly higher than the prevalence from central Ethiopia, 22.9% [16], Nigeria, 6.7% [10], Pakistan, 11.1% [24], and northeastern China, 3.0% [25], while it is lower than the seroprevalence of 52.6% using MDAT and 56% with ELISA from Nazareth, Ethiopia [19].

In goats, the percentage of* T. gondii* infection is in consistence with the reported prevalence of 24.1% using MDAT and 25.9% by ELISA in Nazareth, Ethiopia [18], and 27.9% in Thailand [26] and higher than 11.6% [16] and 15.48% [27] in Central Ethiopia. However, on the contrary, it was lower than the reported prevalence of 35% in Debre Berhan, Ethiopia [16], and 44.3% in Egypt [28].

The seroprevalence of* T. gondii* infection in cattle observed in this study is comparable with the reported seroprevalence of 6.6% from Central Ethiopia [16], 15.77% in Iran [29], and 13% in Tanzania [11]. However, the current value is higher than the reported seroprevalence of 2.68% in Brazil [30] and 3.92% in Algeria [31]. In contrast, it is lower than that of the determined seroprevalence of 22.3% in Thailand [32], 32% in Sudan [12], and 43.5% in Pakistan [33].

In the present study, the proportion of camels positive for anti-*T. gondii* antibodies (14.4%) at animal level is in consistence with the proportion in Sudan (20%) [12], while it is higher than that of 3% in China [34].

The flock/herd level seroprevalence in sheep, goats, cattle, and camels in the current study was high. The flock seroprevalence recorded in sheep is like that of the previously reported value of 70.48% in East and West Shewa zones of Oromia Region, Ethiopia [15], and 58.89% in Algeria [31]. In goats, the flock seroprevalence was higher than that of 45.17% reported by Swai and Kaaya [13] in Tanzania. The variation in seroprevalence of* T. gondii* infection among domestic ruminants in the current study from those previously reported in Ethiopia and elsewhere in the world might be attributed to differences in geographical location, animal management practices, and the sensitivity and specificity of serological diagnostic tests used for detecting the infection.

In this study, a significant difference in* T. gondii* seropositivity was observed between the two sex groups of sheep. Female sheep were 2.63 times more infected than males and those that drank from pond and pipe water sources were 4.25 and 9.57 times more infected than those that drank from mixed water sources, respectively. This finding was in accordance with that reported by Ramzan et al. [24]. The increased susceptibility of females might be associated with their lower immunologic resistance in certain periods of their lives [35]. In contrast, Silva et al. [36] and Lashari and Tasawar [37] observed that higher seroprevalence in male sheep than in females is attributed to androgen production lowering their immunity.

In goats, those >1 year old were 3.45 times more infected than those ≤1 year old. Besides, goats that drank pond and pipe water had 6.03 times and 11.61 times more chance of acquisition of* T. gondii* infection, respectively. Likewise, cattle that obtained drinking water from ponds and pipes were 5.60 and 10.68 times more infected with* T. gondii* compared with those that used other water sources. Camels > 4 years old were 2.49 times more infected compared with those ≤4 years old.

The progressive increase in seroprevalence with age seen in sheep, goat, cattle, and camel indicates sustained exposure to the* T. gondii* infection in the environment [6, 19]. Moreover, in the present study, a significantly higher* T. gondii* infection was found in goats > 1 year old and camel > 4 years old compared with the remaining age groups. This may be due to the fact that animals that lived longer might be more likely exposed to the infectious agent from different sources [19, 26].

The observed increased risk of infection in sheep, goats, and cattle that were given pipe water might be explained by the presence of several roaming cats capable of contaminating the pipe water source with infective* T. gondii* oocysts. Silva et al. [36] and Tenter [38] suggested that few cats are sufficient to contaminate a wide field area in short time, since one infected cat sheds millions of oocysts. In addition, watering troughs and the storage of animal feeds stored outdoor which are accessible to cats might be contributed to the heightened seropositivity of* T. gondii* infection among domestic ruminants.

Consistent with the present finding, Gebremedhin et al. [15] reported high risk of infection in sheep given pipe water. However, contrary to our finding, Pinheiro et al. [5] observed increased chances of infection in animals living on properties with running water systems than those living with stagnant water sources. The similarities and differences might be attributed to the resemblance and variations in agroecological situation and rate of contamination of the water properties by infective oocysts.

## 5. Conclusion

It could be concluded that* T. gondii* infection is more common and widespread among domestic ruminants found in the study area. Sex, age, and water source act as risk factors for* T. gondii* infection. Thus, the higher seroprevalence encountered in these animal species used as a food source revealed the potential risk of* T. gondii* infection presented to people through consumption of their meat. Therefore, awareness of people on ways of transmission and prevention of* T. gondii* infection should be raised through education and further study should be conducted to explore the impact of the disease on food animal production.

## Figures and Tables

**Table 1:** Seroprevalence of *T. gondii* infection in domestic ruminants.

Species	Animal level				Herd/flock level			
	*N*	No. of positive	Percentage (%)	95% CI	*N* ^*∗*^	No. of positive	Percentage (%)	95% CI
Sheep	332	112	33.73	28.66–39.10	102	62	60.78	50.62–70.31
Goats	410	113	27.56	23.29–32.16	120	67	55.83	46.48–64.89
Cattle	326	35	10.74	7.59–14.61	82	19	23.17	14.56–33.80
Camels	292	42	14.38	10.57–18.94	47	25	53.19	38.08–67.89

Total	1360	302	22.2	20.02–24.51	351	173	49.29	43.94–54.65

*N*: number of animals tested; No.: number; *N*^*∗*^: number of herds/flocks tested; CI: confidence interval (CI).

**Table 2:** Analysis of risk factors related to *T. gondii* seropositivity in sheep at animal level (*n* = 332).

Variables	Category	Number tested	Positive (%)	Crude OR (95% CI)	Adjusted OR (95% CI)	*P* value
District	Haramaya	95	25 (26.3)	1.00 (ref.)	1.00 (ref.)	
	Gursum	143	44 (30.8)	1.24 (0.70–2.22)	0.69 (0.35–1.34)	0.270
	Babille	94	43 (45.7)	2.36 (1.28–4.35)	0.95 (0.46–1.94)	0.881

Environment	Rural	253	58 (22.9)	1.00 (ref.)	—	—
	Urban	79	54 (68.4)	7.26 (4.16–12.68)	—	—

Sex	Male	68	11 (16.2)	1.00 (ref.)	1.00 (ref.)	—
	Female	264	101 (38.3)	3.21 (1.61–6.41)	2.63 (1.18–5.88)	0.019^*∗*^

Age	≤1 year	39	9 (23.1)	1.00 (ref.)	1.00 (ref.)	
	>1 year	293	103 (35.2)	1.81 (0.83–3.95)	1.15 (0.45–2.91)	0.772

Flock size	Small	270	89 (33.0)	1.00 (ref.)	—	—
	Large	62	23 (37.1)	1.20 (0.68–2.13)	—	—

Cats contact	No	207	62 (30.0)	1.00 (ref.)	1.00 (ref.)	—
	Yes	125	50 (40.0)	1.56 (0.98–2.48)	1.28 (0.74–2.20)	0.371

Water source	Mixed^†^	198	34 (17.2)	1.00 (ref.)	1.00 (ref.)	
	Pond^‡^	55	24 (43.6)	3.73 (1.95–7.14)	4.25 (2.15–8.38)	<0.001^*∗*^
	Pipe water	79	54 (68.4)	10.42 (5.71–19.00)	9.57 (5.00–18.33)	<0.001^*∗*^

House type	Pen	24	3 (12.5)	1.00 (ref.)	—	—
	Both	207	48 (23.2)	2.11 (0.60–7.39)	—	—
	Fence	101	61 (60.4)	10.68 (2.99–38.15)	—	—

Mixed^†^: river, stream water, and well; Pond^‡^: stagnant water; OR: odds ratio; CI: confidence interval. ^*∗*^Significant.

**Table 3:** Logistic regression analysis of risk factors with *T. gondii* seropositivity in goats at animal level (*n* = 410).

Variables	Category	Number tested	Positive (%)	Crude OR (95% CI)	Adjusted OR (95% CI)	*P* value
District	Haramaya	121	22 (18.2)	1.00 (ref.)	1.00 (ref.)	—
	Gursum	176	61 (34.7)	2.39 (1.37–4.16)	0.52 (0.20–1.36)	0.185
	Babille	113	30 (26.6)	1.63 (0.87–3.03)	0.15 (0.05–0.48)	<0.001^*∗*^

Environment	Rural	340	79 (23.2)	1.00 (ref.)	—	—
	Urban	70	34 (48.6)	3.12 (1.83–5.31)	—	—

Sex	Male	93	16 (17.2)	1.00 (ref.)	1.00 (ref.)	–
	Female	317	97 (30.6)	2.12 (1.18–3.82)	1.65 (0.86–3.18)	0.134

Age	≤1 year	63	6 (9.5)	1.00 (ref.)	1.00 (ref.)	—
	>1 year	347	107 (30.8)	4.24 (1.77–10.12)	3.45 (1.34–8.90)	0.010^*∗*^

Breed	Hararghe highland	29	5 (17.2)	1.00 (ref.)	1.00 (ref.)	—
	Long ear Somali	349	95 (27.2)	1.80 (0.67–4.84)	1.10 (0.36–3.39)	0.863
	Undetermined	32	13 (40.6)	3.28 (0.99–10.84)	2.77 (0.68–11.31)	0.155

Flock size	Small	324	95 (29.3)	1.00 (ref.)	1.00 (ref.)	—
	Large	86	18 (20.9)	0.64 (0.36–1.13)	0.85 (0.45–1.60)	0.607

Cats contact	No	270	70 (25.9)	1.00 (ref.)	—	—
	Yes	140	43 (30.7)	1.27 (0.81–1.99)	—	—

Water source	Mixed^†^	188	29 (15.4)	1.00 (ref.)	1.00 (ref.)	—
	Pond^‡^	152	50 (32.9)	2.69 (1.60–4.52)	6.03 (2.42–15.05)	<0.001^*∗*^
	Pipe water	70	34 (48.6)	5.18 (2.80–9.56)	11.61 (4.35–30.95)	<0.001^*∗*^

House type	Pen	27	5 (18.5)	1.00 (ref.)	—	—
	Both	292	73 (25.0)	1.47 (0.54–4.01)	—	—
	Fence	91	35 (38.5)	2.75 (0.95–7.93)	—	—

Mixed^†^: river, stream water, and well; Pond^‡^: stagnant water; OR: odds ratio; CI: confidence interval. ^*∗*^Significant.

**Table 4:** Logistic regression analysis of risk factors with *T. gondii* seropositivity in cattle at animal level (*n* = 326).

Variables	Category	Number tested	Positive (%)	Crude OR (95% CI)	Adjusted OR (95% CI)	*P* value
District	Haramaya	87	14 (16.1)	1.00 (ref.)	1.00 (ref.)	—
	Gursum	153	9 (5.9)	0.33 (0.13–0.79)	0.19 (0.06–0.59)	0.004^*∗*^
	Babille	86	12 (14.0)	0.85 (0.37–1.95)	1.60 (0.49–5.16)	0.434

Environment	Rural	303	30 (9.9)	1.00 (ref.)	—	
	Urban	23	5 (21.7)	2.53 (0.88–7.30)	—	

Sex	Male	97	11 (11.3)	1.00 (ref.)	—	
	Female	229	24 (10.5)	0.92 (0.43–1.95)	—	

Age	≤1 year	34	3 (8.8)	1.00 (ref.)	—	
	>1–5 years	186	19 (10.2)	1.18 (0.33–4.21)	—	
	>5 years	106	13 (12.3)	1.44 (0.39–5.40)	—	

Herd size	Small	218	27 (12.4)	1.00 (ref.)	1.00 (ref.)	—
	Large	108	8 (7.4)	0.57 (0.25–1.29)	0.35 (0.13–0.97)	0.044^*∗*^

Cats contact	No	223	19 (8.5)	1.00 (ref.)	1.00 (ref.)	—
	Yes	103	16 (15.5)	1.97 (0.97–4.02)	2.23 (0.86–5.77)	0.097

Water source	Mixed^†^	234	14 (6.0)	1.00 (ref.)	1.00 (ref.)	—
	Pond^‡^	69	16 (23.2)	4.75 (2.18–10.32)	5.60 (2.12–14.78)	<0.001^*∗*^
	Pipe water	23	5 (21.7)	4.37 (1.41–13.49)	10.68 (2.23–51.22)	0.003^*∗*^

House type	Barn	29	5 (17.2)	1.00 (ref.)	1.00 (ref.)	—
	Both	246	23 (9.4)	0.50 (0.17–1.42)	0.23 (0.05–0.99)	0.049
	Fence	51	7 (13.7)	0.76 (0.22–2.67)	0.42 (0.08–2.32)	0.318

Mixed^†^: river, stream water, and well; Pond^‡^: stagnant water; OR: odds ratio; CI: confidence interval. ^*∗*^Significant.

**Table 5:** Analysis of risk factors with *T. gondii* seropositivity in camels at animal level (*n* = 292).

Variables	Category	Number tested	Positive (%)	Crude OR (95% CI)	Adjusted OR (95% CI)	*P* value
District	Gursum	95	13 (13.7)	1.00 (ref.)	—	—
	Babille	197	29 (14.7)	1.09 (0.54–2.20)	—	—
Sex	Male	73	11 (15.1)	1.00 (ref.)	—	—
	Female	219	31 (14.2)	0.93 (0.44–1.96)	—	—
Age	≤4 years	110	9 (8.2)	1.00 (ref.)	1.00 (ref.)	—
	>4 years	182	33 (18.1)	2.49 (1.14–5.42)	2.49 (1.14–5.45)	0.022^*∗*^
Herd size	Small	203	30 (14.8)	1.00 (ref.)	—	—
	Large	89	12 (13.5)	0.90 (0.44–1.85)	—	—
Cat contact	No	246	32 (13.0)	1.00 (ref.)	1.00 (ref.)	—
	Yes	46	10 (21.7)	1.86 (0.84–4.10)	1.87 (0.84–4.18)	0.127
Water source	Mixed^†^	215	30 (14.0)	1.00 (ref.)	—	—
	Pond^‡^	77	12 (15.6)	1.14 (0.55–2.35)	—	—

Mixed^†^: river, stream water, and well; Pond^‡^: stagnant water; OR: odds ratio; CI: confidence interval. ^*∗*^Significant.
